# Comparison of In Vitro Estrogenic Activity of *Polygoni multiflori* Radix and *Cynanchi wilfordii* Radix via the Enhancement of ERα/β Expression in MCF7 Cells

**DOI:** 10.3390/molecules28052199

**Published:** 2023-02-27

**Authors:** Reshmi Akter, Dong Uk Yang, Jong Chan Ahn, Muhammad Awais, Jinnatun Nahar, Zelika Mega Ramadhania, Jong Yun Kim, Gyong Jai Lee, Gi-Young Kwak, Dong Wook Lee, Byoung Man Kong, Deok Chun Yang, Seok-Kyu Jung

**Affiliations:** 1 Graduate School of Biotechnology, College of Life Sciences, Kyung Hee University, Yongin-si 17104, Gyeonggi-do, Republic of Korea; 2 Hanbangbio Inc., Yongin-si 17104, Gyeonggi-do, Republic of Korea; 3 SaeromHanbang R&D Center, 76, Cheonseok-gil, Geumcheon-myeon, Naju-si 520010, Jeollanam-do, Republic of Korea; 4 SD Leo R&D Center, 9-16, Yeonmujang 5-gil, Seongdong-gu, Seoul 100011, Republic of Korea; 5 Department of Oriental Medicinal Biotechnology, College of Life Sciences, Kyung Hee University, Yongin-si 17104, Gyeonggi-do, Republic of Korea; 6 Department of Horticulture, Kongju National University, Yesan 32439, Republic of Korea

**Keywords:** *Poligonum multiflorum*, *Cynanchum wilfordii*, phytoestrogen, menopause, estrogenic-activity, inflammation

## Abstract

Postmenopausal women experience several symptoms, including inflammation and a sharp rise in oxidative stress caused by estrogen deprivation. Although estrogen replacement therapy (ERT) is generally regarded as an effective treatment for menopause, it has been used less frequently due to some adverse effects and high costs. Therefore, there is an immediate need to develop an effective herbal-based treatment that is affordable for low-income populations. Acordingly, this study explored the estrogen-like properties of methanol extracts from *Cynanchum wilfordii* (CW) and *Poligonum multiflorum* (PM), two important medicinal plants in Republic of Korea, Japan, and China. Due to the similar names and morphologies of these two radixes, they are frequently confused in the marketplace. Our previous colleagues discriminated between these two plants. In this study, we investigated the estrogenic activity of PM and CW using several in vitro assays with their possible mechanism of action. First, their phytochemical contents, such as gallic acid, 2,3,5,4′-tetrahydroxystilbene-2-O-glucoside (TSG) and emodin, were quantified using high-performance liquid chromatography (HPLC). Secondly, estrogen-like activity was assessed utilizing the well-known E-screen test and gene expression analysis in estrogen receptor (ER)-positive MCF7 cells. ROS inhibition and anti-inflammatory effects were analyzed using HaCaT and Raw 264.7 cells, respectively. Our findings demonstrate that PM extracts significantly increased the expression of the estrogen-dependent genes (ERα, ERβ, pS2) and boosted MCF7 cell proliferation in comparison to CW extracts. Additionally, PM extract demonstrated a significant reduction in reactive oxygen species (ROS) production as well as an enhanced antioxidant profile compared to the CW extract. Further, the PM extract treatment significantly reduced the generation of nitric oxide (NO) in RAW 264.7 cells, a murine macrophage cell line, demonstrating the anti-inflammatory properties of the extract. Finally, this research offers an experimental foundation for the use of PM as a phytoestrogen to minimize menopausal symptoms.

## 1. Introduction

The herbal plants of *Poligonum multiflorum* (PM) from the family Polygonaceae and *Cynanchum wilfordii* (CW) from the family Apocynaceae are widely available in Republic of Korea, Japan, and China and are used as oriental medicine. CW is known as Baekshuoh in Republic of Korea and Beishuwu in China, and PM is called Hashuoh in Republic of Korea and Heshuwu in China [1]. Stilbenes and anthraquinones are the primary components in PM, with 2,3,5,4′-tetrahydroxystilbene-2-O-glucoside (TSG), emodin-8-O-D-glucoside (EMG), and physcion-8-O-D-glucoside (PG) being dominant in PM; the bioactivities of PM are thought to be caused by these molecules [2]. More than 300 substances have been identified from Cynanchum species, with steroids, alkaloids, terpenes, flavonoids, polysaccharides, and steroidal glycosides being the main components [3]. Studies have shown that PM possesses anti-bacterial, anti-inflammation, anti-oxidant, liver protection, bone protection, anti-HIV, anti-diabetic, anti-atherosclerotic, anti-tumor, and anti-cancer activities [2]. CW root has been used in traditional Korean medicine to treat hypertension and geriatric and musculoskeletal diseases, including gray hair, muscle impotence, bone weakness, hypercholesterolemia, and tumors [4]. However, because of their similar morphologies and names, CW and PM are frequently utilized indiscriminately in the Korean herbal medicine market [5]. PM radix is made up of dried root tubers of *P. multiflorum*. In contrast, CW radix is truly an appellative word for the root tubers of *C. wilfordii*, according to the Korean, Japanese, and Chinese pharmacopeias [6]. Traditional methods of authenticating medicinal plants have relied on their morphological characteristics; however, identification can be difficult depending on the growth phases and current environmental circumstances [7]. Several studies have attempted to discriminate between these plant species to standardize their usage as medicine [1,8]. In a previous study by our colleagues, *C. wilfordii*, *C. auriculatum*, and *P. multiflorum* were discriminated through chloroplast genes via multiplex PCR [5].

Menopause is a normal biological stage of a woman’s life, characterized by the cessation of menstruation as a result of estrogen deprivation, which typically happens between the ages of 40 and 58 [9]. Estrogen is a regulatory hormone that plays a crucial role in women’s sexual and reproductive development [10] and is mainly a class of steroids produced by the ovary or placenta. After women enter menopause, estrogen levels decline significantly due to aromatase inhibitors [11]. Menopause triggers several menopause-related conditions, including insomnia [12], osteoporosis [13], metabolic disorders [14], and cardiovascular diseases [15].

Systemic inflammation is fueled by the loss of estrogen during the menopause transition [16]. Menopause is marked by a rise in pro-inflammatory serum indicators (IL1, IL6, TNF-α), an increase in cell sensitivity to these cytokines, a decrease in CD4 T and B lymphocytes, and inflammation development [17]. Estrogen replacement therapy (ERT) has been the gold standard for treating menopause symptoms [18]. However, ERT has multiple side effects, including an increased risk of breast cancer [19]. Continued hormone therapy may increase the risk of ovarian cancer, endometrial cancer, blood clots, strokes, and gallbladder diseases [20]. Due to these adverse effects, research into alternative treatments is critical. Many researchers have found natural herbs attractive to treat menopause and related symptoms.

Natural products are medicinal compounds used to treat innumerable disorders since prehistoric times. The identification of the nutritional values, biological activities, and potential health benefits of natural products and their chemical compounds has increased their acceptance by individuals for treating uncountable diseases in recent years [21]. Moreover, the adverse side effects of synthetic drugs have brought more attention to natural product research.

The present study focuses on a comparative analysis of PM and CW and their effects against menopause and postmenopausal symptoms, such as inflammation; we further investigated the chemical compounds responsible for the estrogen-like activity. To our best knowledge, this is the first study examining the effect of these plants on menopause and related symptoms with underlying mechanisms.

## 2. Result and Discussion

### 2.1. Phytochemical Analysis Using HPLC

Phenolic phytochemicals, the most prevalent family of bioactive molecules, can be found in various plant sources, including fruits, vegetables, and drinks [22]. Plant phenolics include tannins, flavonoids, phenolic acids, lignins, and stilbenes. Gallic acid, also referred to as 3,4,5-trihydroxybenzoic acid, is a phenolic substance found both in a free state and as gallotannin (a component of tannins) [23]. Compared with well-known antioxidant vitamins, phenolic acids have substantially stronger in vitro antioxidant activity [24]. These phytochemicals have gained popularity due to their numerous dietary health benefits and capabilities, including their anti-cancer, anti-allergenic, anti-atherogenic, anti-thrombotic, anti-microbial, anti-inflammatory, cardioprotective, and immunoregulatory characteristics [25]. *P. multiflorum* and *C. wilfordii* contain several phytochemicals [26,27]. However, our results showed that the dry weights of 0.17 ± 0.016 mg/g gallic acid and a trace amount of ellagic acid were found in PM. Neither gallic acid nor ellagic acid was found in CW. TSG (2,3,5,4,-tetrahydroxystilbene-2-O-β-d-glucoside), one of the key active ingredients of PM, possesses antioxidant, anti-inflammatory, anti-tumor, anti-HIV, and liver-protective properties [28]. Emodin ((3-methyl-1,6,8-trihydroxyanthraquinone), another principal constituent of PM, exhibits anti-cancer, anti-inflammatory, anti-viral, anti-bacterial, anti-osteoporotic, anti-diabetic, hepatoprotective, and immunosuppressive activity [29]. Our results showed that 39.01 ± 0.280 mg/g DW and 1.18 ± 0.155 mg/g DW TSG were present in PM and CW, respectively, while 0.84 ± 0.003 mg/g DW of emodin was present in PM and it was not present in CW (Table 1).

**Figure 1:**
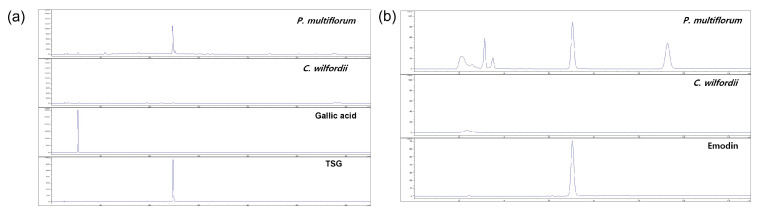
Determination of phytochemical constituents via HPLC analysis. (**a**) Determination of gallic acid and TSG in *P. multiflorum* and *C. wilfordii*. (**b**) Determination of emodin in *P. multiflorum* and *C. wilfordii*.

Additionally, compared to CW, PM contains more gallic acid, TSG, and EG, according to our findings (Figure 1).

### 2.2. Total Phenolic and Total Flavonoid Contents

Natural sources of various phytochemicals, including phenols, flavonoids, alkaloids, glycosides, lignins, and tannins, include plants and plant products. The most prevalent phytochemicals involved in antioxidant activities are phenols and flavonoids found in various fruits, vegetables, and medicinal plants [30]. Flavonoids and phenolics are secondary plant metabolites shielding plant cells from oxidative stress and environmental toxins. They are well-known as antioxidants and have been the subject of interest due to their advantages for human health, including their ability to treat and prevent numerous diseases [31]. Their redox characteristics, crucial in adsorbing and neutralizing free radicals, quenching singlet, triplet oxygen, or degrading peroxides, are thought to be the primary cause of this action. They possess potential effects against inflammation, ulcer, depression, tumor, and cancer [32]. The amounts of phenolic compounds in aqueous extracts of PM and CW are listed in Table 2. The TPC values ranged from 14.03 ± 0.03 to 2.08 ± 0.01 mg/g, represented as gallic acid equivalents (GAE), whereas the TFC contents ranged from 4.81 ± 0.01 to 5.84 ± 0.03 mg/g, expressed as rutin equivalents (RE).

### 2.3. Antioxidant Activity: DPPH and Reducing Power Assays

An antioxidant is a chemical that prevents or delays the oxidative damage that can occur to organisms’ cells by scavenging free radicals, such as peroxide or hydroperoxide, which lowers the risk of developing degenerative diseases [33]. Moreover, numerous severe human diseases, including cancer, Alzheimer’s disease, heart, renal, and liver conditions; fibrosis; atherosclerosis; arthritis; neurological disorders; and aging, may be brought on by abnormal free radical generation [34,35]. Antioxidants are a class of substances that shield cells from free radicals and can slow the onset of illnesses, such as cancer and aging, and boost the immune system [36]. To preserve food and stop the oxidation process, synthetic antioxidants with a neutral flavor have been employed as chemicals for decades but may have carcinogenic effects [37]. Plant antioxidants are crucial to human health because they aid in the body’s ability to combat free radicals and lessen the impact of oxidative stress [38]. To evaluate the antioxidant activity of our extracts, diphenyl-picryl-hydrazine, a stable free radical, was used. The potential of the DPPH free radical to change from violet to yellow depends on its acceptance of a proton donation from the extracts. In the DPPH results, the scavenging capacity of PM and CW was 0.95 ± 0.01 and 0.81 ± 0.01 µg GAE/mg extract, respectively. This result shows that PM has slightly higher antioxidant efficacy than CW.

A reducing power test can be used to determine the ability of Fe^3+^ to transfer to Fe^2+^, which subsequently combines with FeCl_3_ to generate the blue (Fe^3+^)_4_[Fe^2+^(CN)_6_]_3_ complex, which has an absorption peak at 700 nm. The ability of the sample to transport electrons was associated with reducing power. The enhanced absorbance suggested an increase in the reducing power of the plant extract [39]. The antioxidant capacity of PM and CW was 3.37 ± 0.01 and 1.80 ± 0.10 µg GAE/mg extract, respectively. These findings suggested that PM extracts showed notable antioxidant properties because of the number of phytochemicals (Table 3).

### 2.4. The Proliferation of Human MCF-7 Cells

Estrogens are well-known for promoting cellular growth. Phytoestrogens influence and enhance estrogen action reciprocally. Phytoestrogens have an estrogen-like impact when estrogen levels are low; when levels are high, they display antiestrogenic activity by competitively binding to estrogen receptors [40]. MCF-7 cells, an estrogen-sensitive cell line, proliferate when exposed to estrogen-like compounds [41,42]. This characteristic may be used to identify whether a chemical is an estrogen because the proliferative effect of natural estrogen is thought to be the hallmark of estrogen action [43]. The MCF-7 cell proliferation assay measures how the cell reacts to an estrogenic or an antiestrogenic substance through the ER-mediated pathway [44]. PM and CW extracts were investigated for the ability to increase the cell proliferation of estrogen-dependent MCF-7 cells. The potentiality of PM and CW extracts to boost the proliferation of estrogen-dependent MCF-7 cells was examined by an E-screen assay.

**Figure 2:**
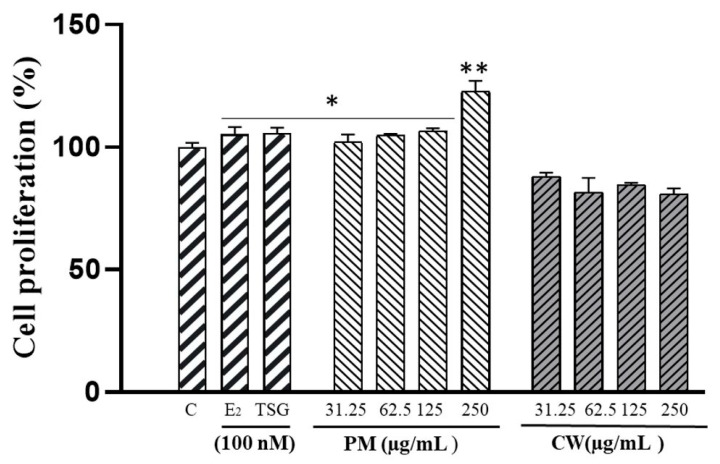
Estrogenic activity of *P. multiflorum* (PM) and *C. wilfordii* (CW) extracts at various concentrations (31.25−250 µg/mL) using an MCF-7 cell proliferation assay. Cell proliferation was determined using MTT assay and is expressed relative to 17β-estradiol (E_2_) at 100 nM. Reported data are the M ± SD of three independent experiments with four replicates each and are expressed as a percent of the response with a control. * *p* < 0.05, ** *p* < 0.01 vs. control (C).

The results revealed a considerable increase in cell proliferation caused by PM extracts (31.25–250 µg/mL) (Figure 2). However, there was no proliferative effect from the CW sample. E_2_ was used as a positive control since it considerably boosted the proliferation of ER-positive MCF-7 cells. Stilbenes such as 2,3,5,4′-tetrahydroxystilbene-2-O-β-D-glucoside (TSG) is a potent phytoestrogen group. A previous study showed that TSG positively affected MCF7 cell proliferation [45]. In our study, we investigated the proliferative effect of TSG on McF7 cells, as PM contains a higher amount of TSG. Additionally, a prior study showed that emodin and emodin 8-O-b-d-glucopyranoside boosted MCF-7 proliferation from 1 to 10 mM [46].

### 2.5. Effects of Plant Extracts on Cell Viability

**Figure 3:**
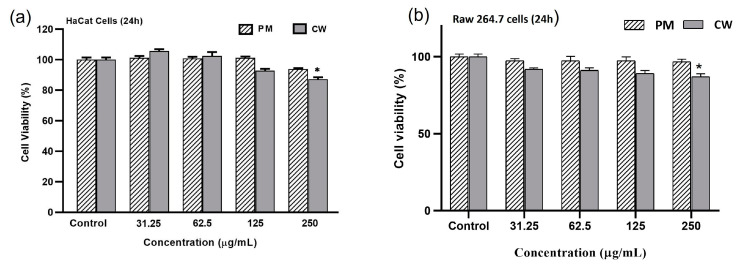
Effects of different concentrations of *P. multiflorum* (PM) and *C. wilfordii* (CW) on viability of (**a**) HaCaT and (**b**) Raw 264.7 cells. Adherent cells seeded in 96-well plates were incubated with various concentrations of PM and CW (31.25–250 µg/mL) for 24 h. Cell viability was determined by MTT assay as described in the Materials and Methods section. Each set of data represents the mean of the triplicate experiment M ± SD. A significant difference between the groups was calculated using a two-tailed Student’s *t*-test. * *p* < 0.05 vs. control is used to represent a significant difference in cell viability of the sample compared with a non-treated control group.

The MTT test was used to measure the vitality of RAW 264.7 and the human keratinocyte cell line (HaCaT) to detect the cytotoxic effect of the PM and CW extracts. As shown in the results (Figure 3a,b), there was no discernible change in the viability of the cells between the control group and the cells treated with 31.25–250 µg/mL PM and CW extract in RAW 264.7 cells; however, 250 µg/mL CW showed slight cytotoxicity in both cells. Based on this result, we selected 200 µg/mL for further experiments.

### 2.6. Effect on Lipopolysaccharide-Induced Nitric Oxide (NO) Production

**Figure 4:**
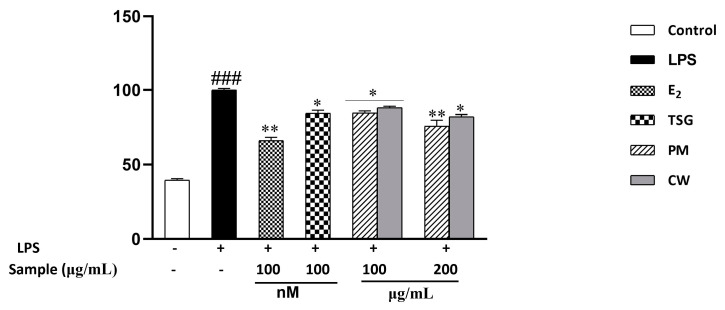
Effects of *P. multiflorum* (PM) and *C. wilfordii* (CW) on NO suppression. After 1 h of pretreatment with PM and CW extracts, RAW 264.7 cells were stimulated with LPS (1 g/mL) for 24 h. Nitrite concentrations were tested as described in the Materials and Methodology. Each data set represents the mean of the triplicate experiment M ± SD. ^###^ *p* < 0.001 compared with control, * *p* < 0.05, ** *p* < 0.01, compared with the group receiving LPS treatment. (+) = with, (−) =without.

In response to inflammation or damage, inducible nitric oxide synthase (iNOS) produces more significant quantities of NO, a signaling molecule crucial to the inflammatory response. In the initial phases of the inflammatory response, macrophages play a pivotal role [47]. Lipopolysaccharide (LPS) activates macrophages, and the production of proinflammatory mediators, such as NO, rises [48]. In treating inflammatory illnesses, using NO inhibitors is an effective therapeutic strategy [49]. In addition, estrogen exerts an anti-inflammatory effect, and deprivation of estrogen levels may increase the risk of inflammation [50]. We investigated the inhibitory effects of PM and CW on nitric oxide generation produced 1 h before and 24 h after applying LPS. Because NO is highly unstable in biological environments and quickly oxidizes to nitrite, the nitrite level in the culture medium was chosen as a measure of NO generation. L-NMMA, a typical nitric oxide inhibitor [51], served as the positive control. Figure 4 demonstrates that NO generation is significantly increased in LPS-treated cells compared with PM- and CW-treated LPS-induced cells. Additionally, antioxidants play significant roles in redox pathways by shielding the cell from inflammatory and apoptotic processes.

Moreover, prior research has demonstrated that flavonoids and phenolics can reduce inflammation by reducing intracellular cytokines and NO production [52,53]. Moreover, previous studies suggested that both TSG and emodin exhibited potential anti-inflammatory effects via inhibiting NO output [54,55]. As PM demonstrated notable flavonoids, phenolics, TSG, and emodin, it also demonstrated more excellent NO generation defense than CW. We also investigated the effect of estradiol against NO production and found that E_2_ significantly inhibited NO levels.

### 2.7. Suppression of Elevated Levels of Reactive Oxygen Species (ROS)

The production of ROS and its eradication by the cellular antioxidant system are balanced in cells under normal circumstances [56]. In postmenopausal women, oxidative stress is increased due to decreased estrogen availability [57]. The overproduction of ROS can harm a cell’s oxidative health by destroying the structural integrity of the cell [58]. H_2_O_2_, a precursor to many radicals, can increase cell ROS levels by piercing the cell membrane [59]. Among the various types of human cells, epidermal keratinocytes reside in the skin’s outermost layer and are constantly exposed to external stimuli, such as UV radiation and H_2_O_2_. As a result, these cells have self-protective functions against environmental threats such as oxidative stress. Although there have been few comparative studies with cells from other organs, epidermal keratinocytes can be considered a type of cell that can compete with ROS. The endogenous redox regulation system in keratinocytes is highly organized regarding the redox state that occurs in response to external stimuli [60]. Pro-inflammatory cytokines were activated through the Mitogen-activated protein kinases (MAPKs) signaling pathway. Thus, the ROS generation using HaCat cells can demonstrate the antioxidant model in general, related to the activation of several signaling pathways, including inflammatory and estrogens [61]. Understanding ROS regulation in metabolic inflammation and estrogen signaling pathways may provide the basis for developing therapeutic strategies for managing metabolic dysfunctions [62]. To investigate in vitro antioxidant potential, 500 µM H_2_O_2_ was first used to stimulate ROS formation in the HaCaT cells before they were treated with PM and CW extracts.

**Figure 5:**
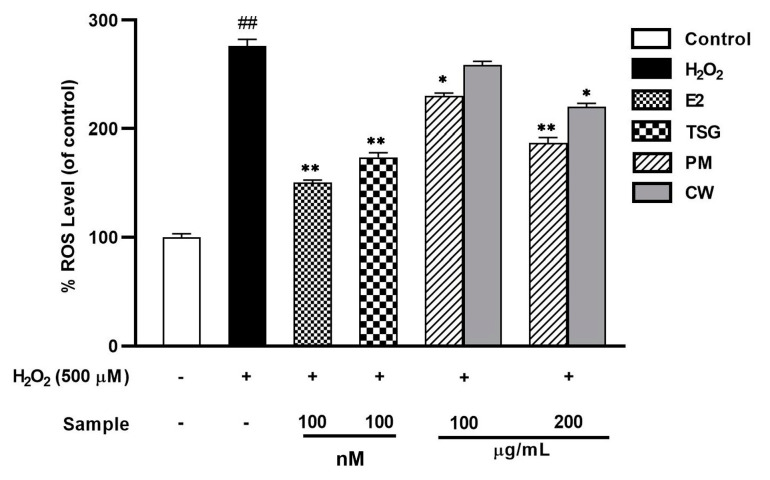
Inhibition of reactive oxygen species (ROS) generation by *P. multiflorum* (PM) and *C. wilfordii* (CW) in H_2_O_2_-induced HaCaT cells was determined using DCFDA. Data are expressed as a percentage of control. ^##^ *p* < 0.01 compared with control, * *p* < 0.05, ** *p* < 0.01 compared with the H_2_O_2_.

Using the fluorescent probe DCFH-DA, the influence of intracellular ROS levels in the HaCaT cells was examined (Figure 5). After H_2_O_2_ stimulation, HaCaT cell ROS levels and fluorescence intensity considerably increased, while both PM and CW lowered the fluorescence intensity. Because estrogens bind to estrogen receptors and use intracellular signaling pathways to up-regulate the production of antioxidant enzymes, they have antioxidant characteristics [63]. We also determined the effect of estrogen on ROS generation, and the result showed that E_2_ exhibited significant ROS inhibition. Up to 200 µg/mL, PM showed a significant ROS inhibitory effect when compared with CW. Polyphenols and flavonoids stop the production of intracellular ROS and shield cells from oxidative damage [64]. PM contains abundant polyphenols and flavonoids and might help prevent HaCaT cells from oxidative damage caused by H_2_O_2_.

### 2.8. Estrogen Receptor mRNA Expression and Estrogenic Activity in Human MCF-7 Cells

The physiological effects of estrogenic substances are significantly modulated by the estrogen receptor subtypes (ERα and ERβ) [65]. The natural estrogen 17β-estradiol (E_2_) has a high affinity for binding to both ER-α and ER-β. Similar ligand-binding specificities are shared by dietary phytoestrogen as they share structural similarities with synthetic estrogen [66]. ER is primarily expressed in the uterus, ovary, breast, kidney, bone, and liver. ER is also found in the ovary, colon, central nervous system, heart, lung, and prostate [67]. Isoflavones, stilbene, coumestan, and lignan are four phenolic chemicals categorized as phytoestrogens [68]. TSG boosted ER expression in MCF-7 cells. Furthermore, TSG reduced estrogen deficiency-induced osteopenia in animal models of osteoporosis caused by ovariectomy [69].

**Figure 6:**
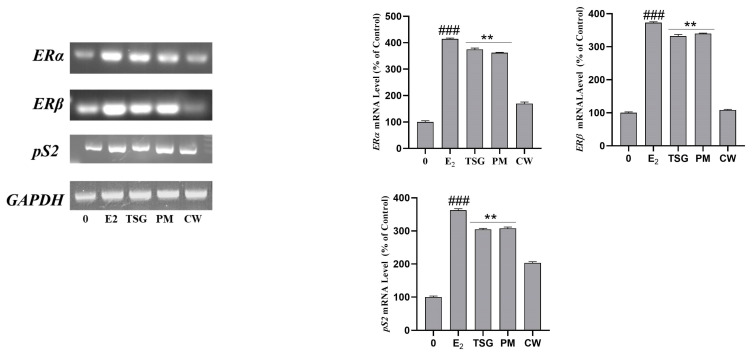
Effect of Pomegranate on the transcriptional activation of the ERα, ERβ, and pS2 genes in MCF7 cells. MCF7 cells were treated with *P. multiflorum* (PM) and *C. wilfordii* (CW) for 24 h. Total RNAs were extracted, and the mRNA expression levels were determined by RT-PCR analysis and compared with those of GAPDH. The data shown are representative of the mean values of three independent experiments M ± SD. ** *p* < 0.01 as compared with the group treated with E_2_, and ^###^ *p* < 0.001 as compared with the control.

To investigate the effect of PM and CW extracts on the proliferation-promoting effects along with *ERα* and *ERβ* activation, we used RT-PCR. We focused on *ERα*, *ERβ*, and the estrogen-regulated gene *pS2* found in the breast cancer cell line MCF7. Numerous studies have used the ER-subtype-mediated route to investigate the phytoestrogenic effects of target substances in vitro and animals [70,71]. Our results revealed that PM extract notably increased the expression of *ERα* and *ERβ* in MCF7 cells. Both genes were significantly expressed compared with estradiol (E_2_) expression. We also checked the effect of TSG on all genes (*ERα*, *ERβ*, and *pS2* genes). TSG demonstrated a nearly identical action as estrogen. PM greatly impacted the upregulation of *ERα*, *ERβ*, and *pS2* gene expression, whereas CW had very little influence (Figure 6). Additionally, PM contains a sufficient amount of the overall phenolic and flavonoid content and a notable amount of gallic acid and has displayed a more preferable estrogenic effect than CW. Therefore, the estrogen-like effect of PM extract was mainly mediated via the ER-mediated pathway.

## 3. Materials and Methods

### 3.1. Collection and Preparation of Plant Material Samples

*Polygoni multiflori* Radix and *Cynanchi wilfordii* Radix were purchased from Donguiherb Co., Ltd. (Seoul, Republic of Korea). As described by Nguyen et al., 2021 [72], plant materials were extracted with minor modifications. We added 80% methanol to 1 g dried powder of *P. multiflorum* and *C. wilfordii* roots and extracted the samples for 15 min in an ultrasonic bath three times. The extracted solution from each extraction was combined and evaporated at 45 °C with a rotary evaporator (Eyela, Japan). The extract was then diluted in 5 mL of HPLC-grade MeOH and filtered with a 0.45 µm syringe filter before analysis by HPLC.

### 3.2. Preparation of Standard Solutions

Gallic acid, 2,3,5,4′-tetrahydroxystilbene-2-O-β-D-glucoside (TSG), and emodin were purchased from Sigma-Aldrich (Darmstadt, Germany), Ensolbio Sciences (Daejeon, Republic of Korea), and Extrasynthese (Genay, France), respectively. The individual standard stock solutions of gallic acid, TSG, and emodin were prepared at a concentration of 1000 mg/L. The various concentrations of standard solutions were plotted against the peak area to create a standard curve to quantify the ingredients in the plant materials (Table 4).

### 3.3. High-Performance Liquid Chromatography (HPLC)

High-performance liquid chromatography (HPLC) was performed as previously described [73]; the HPLC conditions for analyzing gallic acid, TSG, and emodin are shown in Table 2. The HPLC system consisted of an Agilent 1260 infinity system equipped with an Agilent 1260 Infinity Quaternary Pump (G1311B), Agilent 1260 Infinity Standard Autosampler (G1329B), Agilent 1260 Infinity Column Thermostat Compartment (G1316A), and Agilent 1260 Infinity Variable Wavelength Detector (G1314F). The ZORBAX Eclipse Plus C18 column (250 mm × 4.6 mm, 5 μm particle size) (Milford, MA, USA) was chosen as a stationary phase. For Gallic acid and TSG analysis, the eluent composition was as follows: (0–8 min, 90–80% B; 8–30 min, 80–55% B; 30–60 min, 55–30% B). Isocratic elution of 0.1% phosphoric acid in water and methanol was chosen to determine emodin in the plant materials. The HPLC analysis conditions are shown in Table 5.

### 3.4. Determination of Total Phenolic and Total Flavonoid Contents

The Folin–Ciocalteu technique was used to determine each sample’s total phenolics and flavonoids following the previous method [74], with a few minor adjustments. After extracting 0.5 g of dried powdered material three times in 20 mL of 80% methanol for 1 h, the filtrate was mixed for evaporation. The crude extract was redissolved in distilled water for further compound analysis. Next, 0.3 mL of each extract was mixed with 1.5 mL of Folin–Ciocalteu reagent in wells of a 96-well microplate to measure total phenolics. The mixture was then incubated for 5 min after being thoroughly shaken. Next, 1 mL of 7.5% Na_2_CO_3_ solution was added, and the sample was left in the dark for 30 min. The absorbance at 715 nm was finally measured. Gallic acid was used as a standard to create a standard curve for evaluating the total phenolic content. Gallic acid equivalent (GAE) was used, and the results were expressed in mole per milligram of extract (µg GAE/mg extract).

The combination reaction of 0.3 mL of each extract, 0.3 mL 5% NaNO_2_, and 0.3 mL 10% AlCl_3_ was used to determine the total flavonoid content. After the mixture was incubated for 6 min, 0.5 mL of 1 N NaOH was added. The absorbance was immediately determined at 510 nm after thoroughly mixing the solution. Rutin was used as a calibration curve to determine the total flavonoid content, and the results are represented as mol rutin equivalent mole per milligram of extract (µg RE/mg extract).

### 3.5. DPPH Scavenging Assay

Using a slightly modified version of the previously published procedure [75], the DPPH method was used to assess the free radical scavenging activity. A 96-well plate was filled with 20 µL of PM and CW extracts and 180 µL of DPPH solution; the plates were then vigorously shaken and incubated for 30 min in the dark at 25 °C. The absorbance was then determined at 517 nm. The following formula was used to calculate the percentage of inhibition of the samples:

(1 − Absorbance of sample/Absorbance of control) × 100.

The reducing power activity of the samples was assessed by mixing 100 µL of the samples with 250 µL of pH 6.6 phosphate buffer and 250 µL of potassium ferricyanide (1%). The mixture was then incubated for 20 min at 40 °C in a water bath. After cooling the mixture, 250 µL of 10% trichloroacetic acid was added. After centrifuging the mixture at 8000 rpm for 10 min, the supernatant was combined with 20 µL of freshly made 0.1% ferric chloride solution and 100 µL of distilled water. The absorbance was calculated at 700 nm. The blank was run without the addition of any extracts. Gallic acid was used as standard, and the results are represented in milligrams of gallic acid equivalents per gram (mg GAE/g DW) of the sample.

### 3.6. Chemical and Reagents for Cell Culture

The human breast cancer cell line (MCF-7) and murine macrophage RAW 264.7 cell line were obtained from American Type Culture Collection (ATCC). Dulbecco’s modified Eagle’s medium (DMEM) was purchased from Daegu (Republic of Korea). Fetal bovine serum (FBS) and P/S were provided by GenDEPOT. Charcoal-dextran and 17β-estradiol were obtained from Sigma (St. Louis, MO, USA).

### 3.7. Cell Culture

MCF7 cells, which proliferate in response to estrogen treatment, were cultured in DMEM (containing 4500 mg/L D-glucose, L-glutamine, sodium pyruvate, and sodium bicarbonate) with 10% fetal bovine serum (FBS) stripped in charcoal-dextran and 1%penicillin–streptomycin (P/S). RAW 264.7 cells were cultured in DMEM containing 10% FBS and 1% P/S. The cells adhered overnight in a humidifier set at 37 °C with a 95% air/5% CO_2_ environment.

### 3.8. E-screen Assay

The E-screen assay was developed based on MCF7 cell proliferative action in response to estrogens [76]. The slightly modified E-screen MCF-7 cell proliferation assay was carried out according to Resende et al. [77]. Briefly, confluent MCF-7 cells were washed with phosphate-buffered saline (PBS) and trypsinized for 1 min. The detached cells were resuspended in DMEM and seeded into 96-well plates at 2 × 10^4^ cells/well. The cells were then incubated in an incubator (37 °C with 5% CO_2_) for 24 h and allowed to adhere. To obtain estrogen-deprived conditions, the medium was aspirated, and an estrogen-free medium was introduced; the medium contained 5% charcoal–dextran-stripped human serum and phenol-red-free DMEM (Invitrogen). The MCF-7 cells were treated with different PM and CW concentrations and cultured for 24 h. In addition, 17β-estradiol and cells without any treatment were used as the positive control.

### 3.9. Cell Proliferation Assay

Cell proliferation was quantified by using 3-(4,5-dimethyl-2-thiazolyl)-2,5-diphenyl-2-H-tetrazolium bromide (MTT) (Sigma-Aldrich, Gillingham, UK) assays. The cells were cultured for 24h. Next, 20 μL of MTT solution (5 mg mL^−1^ stock in PBS pH 7.1, diluted 1:2.5 (*v*/*v*) in assay media) was added to the cells and cells were incubated for 3 h; the medium was replaced with 1 mL dimethyl sulfoxide (DMSO). The absorbance was determined at a wavelength of 570 nm using a microplate reader (Molecular Devices Inc., Sunnyvale, CA, USA). Cell proliferation was represented as a percentage compared to the negative control, which was taken to mean 100% cell proliferation.

### 3.10. Cell Viability Assay

The MTT cell viability assay was used to check for any potential cytotoxicity in the PM, and CW extracts on RAW 264.7 cells. Through mitochondrial succinate dehydrogenase, viable cells transform soluble yellow MTT into an insoluble purple formazan. Cells were seeded in 96-well at 2 × 10^4^ cells/well and incubated for 24 h. The cells were then treated with different concentrations of PM and CW. The supernatant was discarded, and 20 µL MTT solution was added to each well; the cells were incubated for 3 h. Following the steps outlined by [78], the cells were stained with 100 µL of DMSO to turn the insoluble formazan crystals into a colored solution; the cell survival rate was measured at 570 nm using an ELISA reader (Bio-Tek Instruments, Inc., Winooski, VT, USA).

### 3.11. Measurement of Cellular ROS in HaCaT Cells

The degree of reactive oxygen species (ROS) formation can be assessed using a common cell-permeable fluorogenic probe, 2′,7′-dichlorofluorescein diacetate (H2DCFDA). In 96-well cell culture plates, HaCaT cells were seeded at 1 × 10^4^ cells per well and incubated overnight to achieve 100% growth confluency. After 24 h of culture in the mixed medium of the PM and CM extract (200 µg/mL), the HaCaT cells were stimulated with 100 μM H_2_O_2_ for 2 h. DCFH-DA (10 M) solution was added to each well to stain the cells, which were then left to sit in the dark for 30 min. The cells were then washed twice with ice-cold PBS. Finally, using a Spectra Fluor multiwell fluorescence reader (Tecan, Maninder, Austria), the fluorescence emission intensity was measured between 485 and 495 nm, respectively, following a previous procedure [79] with some slight modifications.

### 3.12. Measurement of Cellular NO Production in RAW 264.7 Cells

The NO inhibition by the samples was determined in LPS-stimulated RAW 264.7 cells following the previously reported method [80]. Briefly, the cells were pretreated with PM and CW before being stimulated with 1 g/mL LPS. The cells were then incubated for 24 h in an incubator. Nitrite levels in the media were measured using the Griess reagent; 100 µL of the supernatant was combined with 100 µL of the Griess reagent. The absorbance was determined at 540 nm using a microplate reader (Bio-Tek Instruments, Inc.).

### 3.13. Gene Expression Analysis

MCF-7 cells were plated in 12-well plates at 5 ×10^5^ cells/well. The medium was aspirated, and phenol red- and serum-free DMEM with or without PM and CW (100 µg/mL) were added. After 24 h, the cells were washed, and the total RNA was extracted using TriZol LS reagents (Invitrogen, Carlsbad, CA, USA) before reverse transcription polymerase chain reaction (RT-PCR), followed by the cDNA synthesis using a commercial cDNA synthesis kit (Onebio, Lithuania) was used. The cDNA synthesis process was performed at 42 °C for 1 h, followed by 5 min at 72 °C. The targeted gene was then amplified using the generated cDNA. The list of RT-PCR primers is shown in Table 6.

The following parameters were employed for the PCR amplifications: 94 °C for 5 min for one cycle, followed by 94 °C for 1 min, 56 °C for 30 s, and 72 °C for 1 min for 30 cycles. ImageJ1.30v software was used for data analysis [81]. GAPDH expression was used to standardize the relative gene expression levels.

**Table 6:** List of the Primers used in the study.

Genes	Forward Primers	Reverse Primers	Reference
*ERα*	CCGCTCATGATCAAACGCTCTAAG	GCCCTCTACACATTTTCCCTGGTT	[82]
*ERβ*	TTCCCAGCAATGTCACTAACTT	TTGAGGTTCCGCATACAGA	
*pS2*	AATGGGCAGCCGTTAGGAAA	GCGCCCAATACGACCAAA	

### 3.14. Statistical Analysis

All of the data were expressed as the mean SE of at least three independent experiments. GraphPad Prism was used to conduct statistical analysis (GraphPad Software, La Jolla, CA, USA). Student’s *t*-test and two-way analysis of variance were used to determine the total variations between treated groups and untreated (control) groups (ANOVA). The difference was considered significant at * *p* < 0.05, ** *p* < 0.01, *** *p* < 0.001.

## 4. Conclusions

The current work used HPLC analysis to identify the phytochemicals in PM and CW extracts. The phytochemical of TSG, a significant phytoestrogen, is in substantial amounts in PM extract. Additionally, the PM extracts contained higher levels of total flavonoids and phenolics than CW extracts. PM possessed more antioxidant qualities compared with CW. In contrast to CW, PM dramatically boosted ER receptor expression in both samples. Estrogen deficiency is a significant contributor to inflammation. PM exhibited an anti-inflammatory effect in the RAW 264.7 cell line. In conclusion, *P. multiflorum* showed better estrogenic, ROS inhibition, and anti-inflammatory activities, and *C. wilfordii* showed a weaker effect. These results indicate that although the plants share similar morphology, their pharmacological actions differ. The PM extracts can be a better alternative to reduce postmenopausal symptoms, but should be further evaluated using inflammation in menopause in in vivo. In addition, careful authentication of these plants should be carried out to avoid improperly selecting these medicinal plants in the dried form.

## Figures and Tables

**Table 1:** Analysis of phytochemicals via HPLC.

Samples	Contents (mg/g DW)		
	Gallic Acid	TSG	Emodin
*P. multiflorum*	0.17 ± 0.016	39.01 ± 0.280	0.84 ± 0.003
*C. wilfordii*	ND	1.18 ± 0.155	ND

Mean ± standard error.

**Table 2:** Analysis of total phenolics and flavonoids.

Samples	TPC (µg GAE/mg Extract)	TFC (µg RE/mg Extract)
PM	14.03 ± 0.03 ^a^	4.81 ± 0.01 ^b^
CW	2.08 ± 0.01 ^c^	5.84 ± 0.03 ^a^

Values in the same column followed by a different letter (a–c) are significantly different at *p* < 0.05.

**Table 3:** Potential antioxidant activities of PM and CW.

Samples	In Vitro Antioxidant	
	DPPH (µg GAE/mg Extract)	Reducing Power (µg GAE/mg Extract)
PM	0.95 ± 0.01 ^a^	3.37 ± 0.01 ^a^
CW	0.81 ± 0.01 ^b^	1.80 ± 0.10 ^c^

Values in the same column followed by a different letter (a–c) are significantly different at *p* < 0.05.

**Table 4:** The parameters of the calibration curve of reference standard substrates.

Standard	Solvent	Regression Equations	R_2_	Linearity Range
Gallic acid		y = 14941x + 118.73	0.9997	0.03125–1 mg mL^−1^
TSG	Methanol	y = 1537.6x + 8.8448	0.9998	0.015625–1 mg mL^−1^
Emodin		y = 5667x − 55.038	0.9985	0.015625–1 mg mL^−1^

**Table 5:** HPLC System and Condition for Analysis of Chemical Contents.

System/Condition	Gallic Acid and TSG	Emodin
Flow Rate	1.0 mL/min	1.0 mL/min
Wavelength	260 nm	436 nm
Injection Volume	5 µL	5 µL
Solvents	Gradient eluent	Isocratic eluent:
	A: Methanol	A: 0.1% Phosphoric acid in water
	B: 0.1% Acetic acid in water	B: Methanol
Column Temperature	35 °C	30 °C

## Data Availability

We do not wish to make the data publicly available as further research is being undertaken based on this study.

## Sample Availability

Samples of the compounds are not available from the authors.
